# Modified scoring criteria to improve the accuracy of the home sleep apnea test

**DOI:** 10.1007/s11325-026-03598-y

**Published:** 2026-02-16

**Authors:** Philip Cushman, Amanda Radtke, James Kang, Aaron Burch, Zahari N. Tchopev, Matthew S. Brock, H. Samuel Scheuller

**Affiliations:** San Antonio Uniformed Services Health Education Consortium, Department of Sleep Medicine, Wilford Hall Ambulatory Surgical Center, Joint Base San Antonio, 88Th Medical Group, 3Rd Floor Sleep Lab, 4881 Sugar Maple Dr, Wright-Patterson AFB, Lackland, TX OH 45433 USA

**Keywords:** Sleep, Apnea, Polysomnography, Arousal

## Abstract

**Purpose:**

The Home Sleep Apnea Test (HSAT) has good diagnostic performance for patients with a high pretest probability of moderate to severe obstructive sleep apnea (OSA). However, the false negative rate has been reported as high as 17%. Therefore, the American Academy of Sleep Medicine (AASM) recommends polysomnography (PSG) after a nondiagnostic HSAT (apnea–hypopnea index (AHI) < 5/hr). Our objective was to improve the accuracy of HSATs by using hyperpneas as a surrogate for arousals.

**Methods:**

A retrospective analysis was conducted on patients with non-diagnostic Type 3 HSATs with subsequent PSG. HSATs were re-scored using the AASM recommended hypopnea scoring including using post-hypopnea hyperpneas without relative desaturations as a surrogate for cortical arousals. The new AHI was then compared with the gold-standard PSG.

**Results:**

We identified 68 patients (80.9% male) with a non-diagnostic HSAT and subsequent PSG. 38 patients (55.9%) had an AHI ≥ 5 on PSG. By applying our modified HSAT criteria, 41 (60.2%) of the previously non-diagnostic HSATs had an AHI ≥ 5. The mean difference in AHI between the modified HSAT criteria and PSG was 3.7/hr, compared to 5.9/hr between the original HSAT and PSG. The overall concordance between the modified HSAT criteria and PSG for OSA diagnosis was 89.7% compared to only 44.1% of the original HSATs.

**Conclusions:**

Incorporating a surrogate indicator of a cortical arousal such as a hyperpnea can improve the diagnostic accuracy of the HSAT. Our modified HSAT scoring criteria improved AHI concordance with PSG with fewer false negatives (5%), thereby decreasing the need for repeat testing and saving costs.

## Introduction

The diagnosis of obstructive sleep apnea (OSA) in adults is determined by the apnea–hypopnea index (AHI) demonstrated during gold standard attended in-lab polysomnography (PSG) or a Home Sleep Apnea Test (HSAT) [1]. The HSAT increases access to OSA testing due to its cost, availability for patients to test at home, and ease of provider interpretation [2]. Conversely, its limited data collection, patient directed application, and differences in provider scoring compared to PSG may decrease its accuracy, necessitating repeat testing or missed diagnoses [3–20].

During PSG, the recommended American Academy of Sleep Medicine (AASM) scoring criteria for a hypopnea is a 30% decrease in airflow accompanied by either an arousal or ≥ 3% oxygen desaturation [21]. Arousals during PSG are determined by changes seen on electroencephalography (EEG). Comparatively, hypopneas during an HSAT are determined solely based on ≥ 3% oxygen desaturation per “recommended” scoring (or ≥ 4% by “optional” scoring rule), as EEG is not part of type 3 or 4 HSATs [21]. For the purposes of this study, the term “HSAT” refers to a Type 3 portable monitoring device unless otherwise stated.

It is also important to note that diagnosis of OSA based on the AASM definition of an AHI ≥ 5/hr is often criticized for its limitations, however to date it remains the best studied metric of OSA severity. Other metrics such as hypoxic burden, arousal intensity, biomarkers, or machine learning could be used in the future as we continue to have further research in OSA diagnosis [22].

A systematic review of 59 studies comparing the accuracy of HSATs to PSGs concluded that HSATs showed good diagnostic performance for those with a high pretest probability of moderate to severe OSA [23]. Some other studies, though, have highlighted that HSATs typically underestimate the severity of mild and moderate sleep disordered breathing [3, 4]. The discordance between HSAT and PSG may be even worse among those with a low arousal threshold (LAT) OSA phenotype, which is not accounted for with traditional hypoxemia-based HSAT scoring [5]. Additionally, using desaturation-only criteria (such as used by the Center for Medicare Services for scoring PSGs) results in an underestimated AHI and a 15–20% false negative test rate for OSA compared with the AASM recommended scoring criteria [6, 7]. Therefore, improving the accuracy of the HSAT using a metric other than desaturation has the potential to bridge this gap, especially for the LAT OSA phenotype.

The AASM reports the false negative rate for HSATs to be as high as 17%, and therefore recommends that a PSG should be performed in any patient with an increased risk for OSA whose HSAT is negative [8]. Furthermore, a systematic review found the false positive rate for HSATs to range from 2—31%. The false positive rate for other home testing modalities (Type 4 home-unattended studies) was even higher, ranging from 41–73% [9, 10].

Multiple studies have been conducted using computer algorithms to attempt to improve the diagnostic sensitivity of HSATs for detecting sleep disordered breathing. These algorithms use other signals such as photoplesthysmography (PPG), peripheral-arterial tonometry (PAT), and electrocardiography to provide accurate estimates of total sleep time and REM sleep periods, and also to detect autonomic arousals as a surrogate for cortical arousals [11–17]. While these algorithms do not have the same accuracy as sleep staging and cortical arousal detection using EEG signals, studies have demonstrated that these algorithmic estimates can help improve the diagnostic sensitivity of HSATs [18, 19].

Similarly, Ayappa et al. evaluated the utility of overnight monitoring limited to nasal cannula airflow and oximetry for diagnosis of OSA. Respiratory Disturbance Index (RDI) was compared between full nocturnal PSG, and PSG and ambulatory studies using only airflow and oximetry channels. While their study used fewer channels than a Type 3 HSAT, the diagnostic sensitivity and specificity using a cutoff of 18 events per hour using only flow signals from the ambulatory study was 88% and 92%, respectively [20]. This study indicates that manual scoring in the absence of EEG arousal channels can also reliably identify OSA.

Johnson et al. [24] evaluated the reliability of scoring a respiratory event measure which they titled Flow Limitation/Obstruction With recovery breath (FLOW) and its correlation with traditional EEG based apneas, hypopneas, respiratory-effort related arousals (RERAs), and other arousals. FLOW was scored on PSGs, and a FLOW event was scored if all of the following criteria were met: A. Run of at least 2 breaths that have evidence of obstruction, B. Must be followed by a distinct change in breathing pattern with increased amplitude (recovery breath), C. Meets distance criteria from other events. Evidence of obstruction was determined by specific morphologic criteria the researchers defined rather than using amplitude and time criteria, such as the traditional 30% decrease for 10 s the AASM has defined for hypopnea scoring. They also defined a recovery breath as a post-event breath that is 50% larger than preceding breaths, among other morphologic characteristics such as roundness and smoothness. That study was intended as an initial step in determining feasibility of scoring FLOW, and stated that future studies of reliability testing of FLOW scoring in HSAT studies were needed [24]. The Johnson et al. study highlights that scoring events related to recovery breaths is reliable between scorers, however while 80% of respiratory-related arousals were concurrent with FLOW events in their study, 56% of FLOW events were independent of traditionally scored events. In our study, we aimed to use scoring criteria that more closely aligns with current AASM scoring rules while still including a recovery breath as a surrogate indicator of arousals on HSATs. We hypothesized that these modified scoring rules would improve the accuracy and decrease the false negative rate of HSATs.

## Methods

A retrospective analysis was conducted on 68 patients with non-diagnostic Type 3 HSAT who underwent subsequent PSG. HSATs were conducted on the Respironics Alice NightOne, and PSGs were conducted on the Respironics Alice LDx. All patients who underwent an HSAT were uncomplicated adult patients who presented with signs and symptoms indicating and increased risk of moderate to severe OSA, in accordance with AASM Clinical Practice Guidelines [8]. The initial statistical power analysis concluded that 30 pairs of HSATs and PSGs would be the minimum to be statistically significant (p < 0.05) assuming an effect size AHI of 2.5 events/hr as diagnostically significant. The effect size of 2.5 events/hr was chosen based on the AASM Inter-scorer Reliability Program inter-scorer agreement of approximately 83% (17% deviation from gold standard) [25]. An AHI change of 2.5/hr represents 50% of the normal diagnostic range (AHI 0–4.9/hr) and 17% of mild to moderate ranges (AHI 5–29.9/hr). Therefore, a change > 2.5/hr would be representative of the change of scoring method rather than interscorer concordance. At the start of this study, 68 patients had already completed a non-diagnostic HSAT and subsequent PSG for 2024 in our sleep laboratory, which was more than twice the number determined for the study to be adequately powered.

**Fig. 1:**
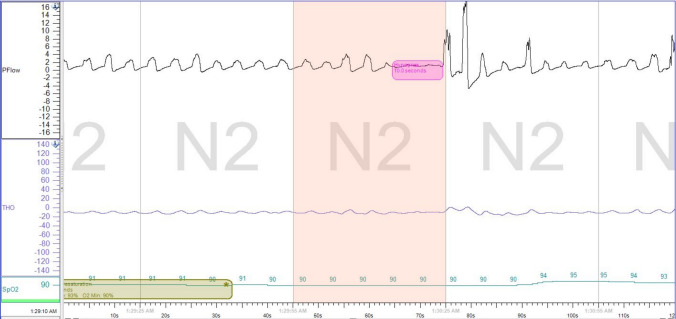
Example of a scored hypopnea by modified scoring criteria: Four HSAT epochs (2 min) showing an example of a hypopnea scored based on the modified scoring criteria. A ≥ 30% decrease in flow for ≥ 10 s (hypopnea) is followed by a breath with ≥ 50% increase in amplitude over baseline breathing (hyperpnea) without a corresponding ≥ 3% oxygen desaturation. HSAT = home sleep apnea testing, N2 = stage N2 sleep, PFlow = respiratory airflow, THO = thoracic respiratory effort, SpO2 = oxygen saturation

Original HSATs were re-scored using the recommended hypopnea scoring rule as defined by the AASM, but also included post-hypopnea hyperpneas without relative desaturations as a surrogate for cortical arousals. A hyperpnea is defined in this study as a breath with ≥ 50% increase in amplitude over baseline breathing following an apparent flow limitation consistent with a hypopnea event. Hypopneas were therefore scored when there was a ≥ 30% decrease in flow for ≥ 10 s followed by either a ≥ 3% oxygen desaturation or a hyperpnea without significant oxygen desaturation (< 3%). Figure 1 shows an example of a hypopnea scored using our modified scoring criteria. The AHI from the modified HSAT scoring criteria (AHI-H-3%M) was compared to the original AHI from the HSAT (AHI-H-3%) as well as the AHI from the PSG (AHI-PSG). Each HSAT was initially re-scored using the above modified criteria by a Sleep Medicine fellow and subsequently verified by an AASM board certified staff physician. Inter-scorer reliability between the fellow and staff physician was good with a mean AHI difference of 1.18 ± 1.19/hr. The AHI-PSG was blinded to the scorers while the HSATs were re-scored.

Demographic and sleep related data were compared for those with and without sleep apnea based on an AHI-PSG ≥ 5 for establishing the diagnosis. Categorical variables were presented as counts and percents and analyzed using Chi-Squared tests. Numerical Variables are presented as means ± standard deviation and analyzed using Student’s t-test. AHI-H-3%M and AHI-PSG were compared using receiving operating curves (ROC) comparing their area under the curve (AUC) estimates. This study received approval from the Institutional Review Board (IRB).

## Results

Demographic information for the entire study sample and by AHI-PSG group are presented in Table 1. Patients were predominantly male (55, 80.9%) and were between ages 22 and 55 (37.7 ± 7.5). The patients had an average body mass index of 29.45 ± 4.2 kg/m^2^. The average Epworth Sleepiness Scale was 12.8 ± 4.8, and the average Insomnia Severity Index was 15.3 ± 6.6.

**Table 1:** Demographic and clinical characteristics of the study population and comparison of patients with and without OSA

	Entire sample (n = 68)	Without OSA (n = 30)	With OSA (n = 38)	*p*
Age, y	37.7 ± 7.5	35.1 ± 8.5	39.8 ± 5.9	.010
Male	55 (80.9%)	21 (67.7%)	34 (89.5%)	.043
Height (in)	69.1 ± 4.0	68.1 ± 4.0	69.8 ± 3.8	.086
Weight (lb)	199.8 ± 32.1	169.1 ± 33.8	202.7 ± 30.3	.402
BMI, kg/m2	29.5 ± 4.2	29.7 ± 4.7	29.2 ± 3.8	.648
ESS	12.8 ± 4.8	13.7 ± 4.6	12.1 ± 4.9	.163
ISI	15.3 ± 6.6	15.8 ± 6.6	15.0 ± 6.7	.645
AHI-H-3%, events/h	2.4 ± 1.2	2.1 ± 1.0	2.7 ± 1.3	.061
AHI-H-3%M, events/h	6.1 ± 3.3	3.8 ± 1.5	8.0 ± 1.3	<.001
AHI-PSG, events/h	7.9 ± 7.7	2.6 ± 1.5	12.2 ± 8.0	<.001

Demographic and clinical characteristics of the study population are compared for patients with and without OSA. Values are presented as mean ± SD and n (%) for categorical values. Comparisons are based on* t* test for groups with equal variances. “Without OSA” is AHI < 5 by PSG scoring, and “With OSA” is AHI ≥ 5 by PSG scoring. *AHI* apnea–hypopnea index, *AHI-H*-3% AHI by original HSAT scoring criteria (3%-only), *AHI-H*-3%M AHI using modified scoring criteria, *AHI-PSG* AHI using PSG criteria (3%), *BMI* body mass index, *ESS* Epworth Sleepiness Scale, *HSAT* home sleep apnea testing, *ISI* Insomnia Severity Index, *PSG * polysomnography

A total of 38 of the 68 non-diagnostic HSATs (55.9%) had an AHI-PSG ≥ 5 on subsequent PSG and were therefore diagnostic of OSA. Of those patients with OSA, the mean AHI-PSG was 12.2/hr. The mean percentage of events scored as hypopneas was 96%, and the mean saturation of peripheral oxygen (SpO2) nadir was 87.6%. Analysis of OSA by severity revealed 29 patients (76.3%) had mild OSA (AHI-PSG 5–14.9/hr), 7 (18.4%) had moderate OSA (AHI-PSG 15–29.9/hr), and 2 (5.3%) had severe OSA (AHI-PSG > 30/hr). Comparatively, 41 of the 68 patients (60.2%) with an AHI-H-3% < 5 had and AHI-H-3%M ≥ 5. Of these, 40 (97.6%) were scored as mild OSA, 1 (2.4%) was scored as moderate OSA, and none were scored as severe OSA.

**Fig. 2:**
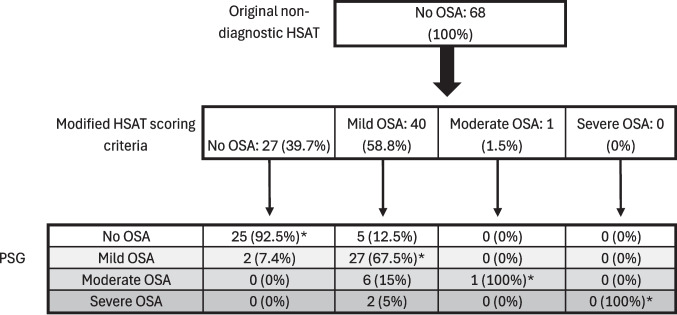
Flow chart of AHI by scoring method: Flow chart depicting change in AHI score based on the scoring method used. OSA severity is based on standard severity cutoffs (mild = ≥ 5–14.9, moderate = ≥ 15–29.9, and severe = ≥ 30). *Indicates categorical agreement between modified HSAT scoring criteria and PSG. AHI = apnea–hypopnea index, HSAT = home sleep apnea testing, OSA = obstructive sleep apnea, PSG = polysomnography

Changes in AHI score by scoring method, as well as concordance between AHI-H-3%M and AHI-PSG are presented in Fig. 2. The overall OSA severity concordance was 77.9%. Concordance was 92.5%, 67.5%, 100%, and 100% for no OSA, mild OSA, moderate OSA, and severe OSA, respectively. Without respect to disease severity, the concordance for presence or absence of OSA was 89.7%.

**Fig. 3:**
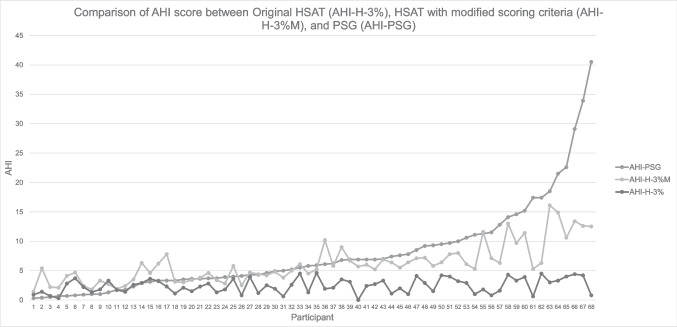
Comparison of AHI score between original HSAT scoring (AHI-H-3%), modified HSAT scoring (AHI-H-3%M), and PSG scoring (AHI-PSG): Participants are plotted in order of increasing AHI-PSG, highlighting the differences in AHI by scoring method used compared to gold-standard PSG. AHI = apnea–hypopnea index, AHI-H-3% = AHI by original HSAT scoring criteria (3%-only), AHI-H-3%M = AHI using modified scoring criteria, AHI-PSG = AHI using PSG criteria (3%), HSAT = home sleep apnea testing, PSG = polysomnography

The AHI for both the AHI-H-3% and the AHI-H-3%M was compared to the AHI-PSG. Figure 3 shows the comparison of AHI between the AHI-H-3%, the AHI-H-3%M, and the AHI-PSG. The mean difference in AHI between the AHI-H-3%M and AHI-PSG was ± 3.7/hr. Comparatively, the AHI-H-3% showed a mean difference in AHI of ± 5.9/hr from the AHI-PSG (p = < 0.0001).

**Fig. 4:**
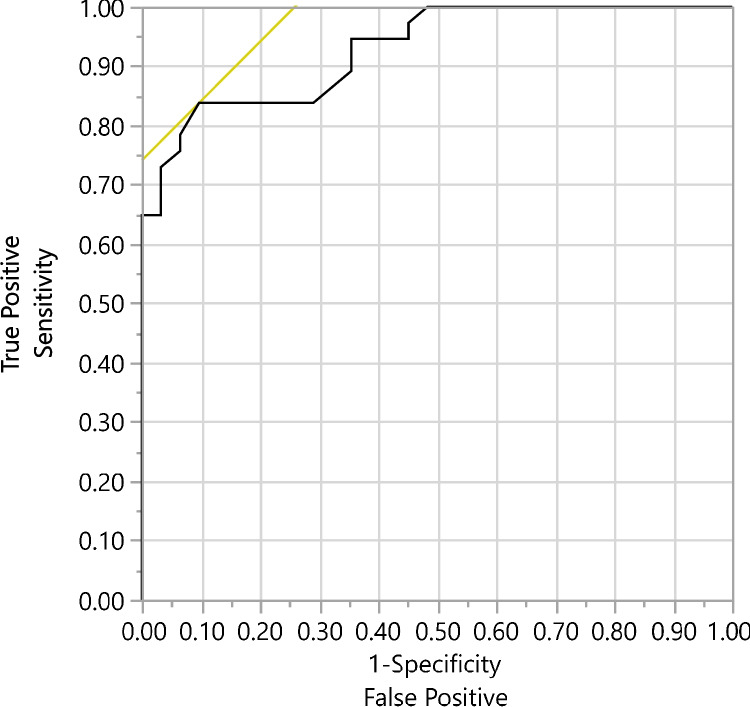
Area Under the Curve (AUC) analysis for the modified HSAT scoring criteria (AHI-H-3%M): Graph depicting AUC analysis for the modified scoring criteria. AUC was 93%. HSAT = home sleep apnea testing

The modified scoring criteria HSAT (AHI-H-3%M) had a false negative rate of 5% for this population and had a false positive rate of 17%. The specificity of the modified scoring criteria HSAT was 83% and the sensitivity was 95% for this population, with an area under the curve (AUC) of 93% (AUC is presented in Fig. 4).

## Discussion

This study demonstrates how incorporating a surrogate indicator of a cortical arousal, in this case a hyperpnea following a flow limitation (hypopnea), can improve the accuracy of the HSAT. Concordance between the AHI-H-3%M and AHI-PSG for OSA severity was good overall (77.9%), and higher for certain severity categories as is shown in Fig. 2. Of the 40 patients with mild OSA by AHI-H-3%M, the OSA severity was underestimated for 8 patients (20%) and had moderate or severe OSA by AHI-PSG. However, when concordance is measured for presence or absence of OSA without regard to disease severity, the concordance between AHI-H-3%M and AHI-PSG is 89.7%. Comparatively, only 44.1% of the AHI-H-3% were concordant with AHI-PSG.

The improvement in accuracy can further be seen in Fig. 3, with AHI-H-3%M more closely aligning with the AHI-PSG compared to the AHI-H-3%, which was statistically significant (p ≤ 0.0001). The AHI-H-3%M had an accuracy within ± 3.7/hr of the AHI-PSG, compared to an accuracy within ± 5.9/h when comparing the AHI-H-3% score to AHI-PSG. This improvement in accuracy also led to improvement in the false negative rate (5%) with the modified scoring criteria (AHI-H-3%M) compared to the 17% false negative rate reported by AASM for the current scoring criteria (AHI-H-3%) [7]. Comparison is made with the false negative rate reported by the AASM for HSATs as only non-diagnostic HSATs (AHI-H-3% < 5) were used in this study, therefore we were unable to calculate a false negative rate for the original HSAT scoring for our population without the true positive value.

The false positive rate (17%) for the modified scoring criteria (AHI-H-3%M) is also similar to what has been reported for current HSAT scoring (AHI-H-3%) in other studies (2–31%) and lower than what is reported for other home testing modalities (Type 4 home-unattended studies, 41–73%) [9, 10]. Additionally, our modified scoring criteria (AHI-H-3%M) had good sensitivity (95%), acceptable specificity (83%), and excellent AUC (93%).

It is important to note that the 5 participants (17%) who were falsely positive based on AHI-H-3%M may still be patients with clinically significant UARS (Upper Airway Resistance Syndrome) type disease. Long-term outcome studies have demonstrated that patients initially diagnosed with UARS remain untreated following initial evaluation and have worsening of symptoms [26, 27]. It is possible that these patients may still benefit from OSA treatment modalities [28].

According to the AASM, the 2025 CMS payment for a Type 3 HSAT (95,806) is $93.16. Comparatively, the payment for an in-lab attended PSG (95,810) is $608.44 [2]. Therefore, the total if PSG is performed after a non-diagnostic HSAT is $701.60, not including the cost for any follow up appointments that may also take place. The AASM recommends that HSAT be considered as the initial sleep study in patients with high pretest probably of OSA [8, 29]. Even so, each year in the United States, approximately 1300 sleep laboratories conduct over 1.1 million PSGs [30]. The potential savings of avoiding PSG for these patients are significant when considering that nearly 30 million Americans are afflicted with OSA and an estimated 80% of cases remain undiagnosed [8].

In a previous cohort of military personnel referred for PSG, 95.2% of the cohort met criteria for LAT phenotype OSA, in which at least 2 of the following criteria are present: (1) > 58.3% of all respiratory events are hypopneas, (2) > 82.5% oxygen saturation nadir, and (3) AHI ≥ 5 events/h but < 30 events/h [5, 31, 32]. All of the patients in our study with AHI ≥ 5/h by AHI-PSG or AHI-H-3%M met criteria for LAT phenotype. Given that most of the obstructive events in patients with LAT phenotype are associated with minimal hypoxemia, arousal-based scoring criteria of hypopneas is essential for accurate calculation of AHI and diagnosis of OSA. This may partially explain why the modified HSAT scoring criteria using a surrogate for arousals was more accurate than traditional HSAT scoring in our population and is likely generalizable to other patients with LAT phenotype OSA.

Our study population also included veterans (Department of Defense beneficiaries, 13.2%). Studies have shown that the prevalence of OSA in veterans is more than twice that of nonveterans, and veterans are diagnosed on average 5 years earlier than nonveterans [33]. The average age of participants in our study was 37.7 and our study also had a male predominance (80.9% male), which aligns with the male predominance reported for this age group [34]. As our study participants were primarily from an active-duty military population (86.8%), the average age and male predominance is expected. Future studies focusing on older populations could improve the generalizability of this scoring method.

An additional limitation of our study is that the HSAT and PSG were performed on different nights and in different environments. Additionally, the time between studies and the body position differences were not part of the study data. Recent studies indicate that night-to-night AHI variability can lead to OSA misdiagnosis and OSA severity misclassification [35–37]. A meta-analysis by Roeder et al. [37] found that while there was no significant difference in mean AHI in two sequential study nights on a group level, there was substantial intraindividual variability. Notably, 49% of participants changed OSA severity class at least once in sequential sleep studies, and 41% of all participants showed changes of respiratory events > 10/h from night-to-night [37]. A follow-on study with HSAT using the modified scoring on the same night as PSG could be performed to eliminate night-to-night AHI differences as a variable.

Overall, our study suggests that our modified scoring criteria for HSATs has the potential to be a more accurate scoring model for HSATs than the current scoring criteria. Our modified criteria resulted in improved diagnostic concordance with PSG, fewer false negatives, and good OSA severity concordance. Implementation of this scoring criteria could therefore lead to fewer PSGs needed after non-diagnostic HSATs, saving both cost and unnecessary testing. Moreover, using this scoring method in combination with other algorithms for estimating autonomic arousals or sleep stage could further improve diagnostic accuracy.

## Abbreviations

AASM: American Academy of Sleep Medicine
AHI: Apnea-Hypopnea Index
AUC: Area Under the Curve
EEG: Electroencephalography
FLOW: Flow Limitation/Obstruction With recovery breath
HSAT: Home Sleep Apnea Test
IRB: Institutional Review Board
LAT: Low Arousal Threshold
OSA: Obstructive Sleep Apnea
PAT: Peripheral-Arterial Tonometry
PPG: Photoplesthysmography
PSG: Polysomnography
RDI: Respiratory Disturbance Index
ROC: Receiving Operating Curve
RERAs: Respiratory-Effort Related Arousals
SpO2: Saturation of Peripheral Oxygen

## Declarations

### Clinical trial

This manuscript does not report on a clinical trial.
